# What pharmacological interventions indicate concerning the role of the perirhinal cortex in recognition memory

**DOI:** 10.1016/j.neuropsychologia.2012.07.034

**Published:** 2012-11

**Authors:** M.W. Brown, G.R.I. Barker, J.P. Aggleton, E.C. Warburton

**Affiliations:** a MRC Centre for Synaptic Plasticity, Department of Physiology and Pharmacology, Bristol University, Bristol, BS8 1TD, UK; b School of Psychology, Cardiff University, Tower Building, Park Place, Cardiff, Wales, CF10 3AT, UK

**Keywords:** Familiarity, Hippocampus, Prefrontal cortex, Consolidation, NMDA, Glutamate, Cholinergic

## Abstract

Findings of pharmacological studies that have investigated the involvement of specific regions of the brain in recognition memory are reviewed. The particular emphasis of the review concerns what such studies indicate concerning the role of the perirhinal cortex in recognition memory. Most of the studies involve rats and most have investigated recognition memory for objects. Pharmacological studies provide a large body of evidence supporting the essential role of the perirhinal cortex in the acquisition, consolidation and retrieval of object recognition memory. Such studies provide increasingly detailed evidence concerning both the neurotransmitter systems and the underlying intracellular mechanisms involved in recognition memory processes. They have provided evidence in support of synaptic weakening as a major synaptic plastic process within perirhinal cortex underlying object recognition memory. They have also supplied confirmatory evidence that that there is more than one synaptic plastic process involved. The demonstrated necessity to long-term recognition memory of intracellular signalling mechanisms related to synaptic modification within perirhinal cortex establishes a central role for the region in the information storage underlying such memory. Perirhinal cortex is thereby established as an information storage site rather than solely a processing station. Pharmacological studies have also supplied new evidence concerning the detailed roles of other regions, including the hippocampus and the medial prefrontal cortex in different types of recognition memory tasks that include a spatial or temporal component. In so doing, they have also further defined the contribution of perirhinal cortex to such tasks. To date it appears that the contribution of perirhinal cortex to associative and temporal order memory reflects that in simple object recognition memory, namely that perirhinal cortex provides information concerning objects and their prior occurrence (novelty/familiarity).

## Highlights

► Perirhinal cortex role in recognition memory. ► Effect of selective pharmacological interventions in specific brain regions on recognition memory. ► Impairments produced by glutamatergic & cholinergic antagonists & compounds targeting intracellular signalling mechanisms. ► Effects on object, object-in-place and temporal order recognition memory.

## 1 Introduction

Although there remains disagreement about the precise role of the perirhinal cortex in recognition memory, there is a consensus that that role (whatever it is) is crucial (see for recent reviews: Brown, Warburton, & Aggleton, 2010; Eichenbaum, Yonelinas, & Ranganath ., 2007; Mayes, Montaldi, & Migo, 2007; Montaldi & Mayes, 2010; Montaldi, Spencer, Roberts, & Mayes, 2006; Squire Wixted, & Clark., 2007; Winters, Saksida, & Bussey, 2008). Recognition memory impairments have been reported following damage to the perirhinal cortex in rats, monkeys and humans (Brown & Aggleton, 2001; Bowles et al., 2007; Clark & Squire, 2010; Eichenbaum et al., 2007; Winters et al., 2008). There is dispute about whether its role can be separated from that of the hippocampus and there remain disagreements concerning whether its role is in familiarity discrimination rather than recollective aspects of recognition memory (Aggleton & Brown, 2006; Eichenbaum et al., 2007; Guderian, Brigham, & Mishkin ., 2011; Montaldi & Mayes, 2010; Montaldi et al., 2006; Murray, Bussey, & Saksida, 2007; Norman, 2010; Squire et al., 2007; Squire & Wixted, 2011; Vann et al., 2009; Vann & Albasser, 2011). Further, it has been argued that the role of perirhinal cortex is determined by its perceptual functions so that its role in recognition memory relates to objects, encompassing both familiarity and recollection relating to objects (e.g., Buckley & Gaffan, 1998, 2006; Montaldi et al., 2006; Murray & Bussey, 1999; Murray et al., 2007; Staresina, Duncan, & Davachi, 2011). Although perirhinal cortex has multimodal inputs, recent findings have indicated a special role in visual rather than auditory or haptic memory (Albasser et al., 2011a,b; Fritz, Mishkin, & Saunders., 2005; Kowalska, Kuśmierek, Kosmal, & Mishkin, 2001; Wan et al., 2001; Winters & Reid, 2010).

Perirhinal cortex is juxtallocortex with a structure that mirrors its transitional nature between neocortex and the archicortex of the hippocampal formation (Burwell, 2001). It has neither the columns of neocortex that allow information concerning an item or feature to be concentrated in one processing module, nor the widely distributed architecture of the hippocampus that potentially allows interconnections between very many distinct items or features, but an intermediate architecture. Such an architecture presumably allows somewhat wider associations than a neocortical column but not the potentially multiple and disparate associations possible within the hippocampus (Brown, 1990). These differing architectures of the perirhinal cortex and hippocampus may provide an underlying reason for the suggested contrasting roles of the perirhinal cortex in recognition memory for items – familiarity for the item and closely linked percepts, ‘within-domain’ as opposed to ‘between-domain’ (Mayes et al., 2007) – and the hippocampus in its broad associative and contextual recognition memory functions – potentially recollection for everything involved with an event (Brown, 1990; Montaldi & Mayes, 2010; Norman, 2010; Norman & O'Reilly, 2003). Differences between the neocortex, hippocampus and perirhinal cortex may also arise from differences in processing capabilities as a result of differences in the pharmacology or biochemistry of the component neurons.

The major focus of this review is to discuss what pharmacological interventions have established concerning the role of the perirhinal cortex in recognition memory processes. As the cellular architecture of the perirhinal cortex differs from that of the hippocampus and neocortex, it should not be assumed that drug effects will always mirror those found in these other cortical regions. The effect upon recognition memory of localised pharmacological interventions in other regions will be discussed chiefly in relation to their implications for perirhinal function. The review will largely concern findings in the rat as this is the species in which most studies have been made. It will concern findings from regionally targeted pharmacological interventions rather than studies of the systemic/global administration of drugs where multiple brain regions may be affected. There have been other recent reviews of pharmacological studies of recognition memory (Dere, Huston, & De Souza Silva., 2007; Lyon, Saksida, & Bussey, 2011; Winters et al., 2008).

### 1.1 Testing rodent recognition memory

**Fig. 1:**
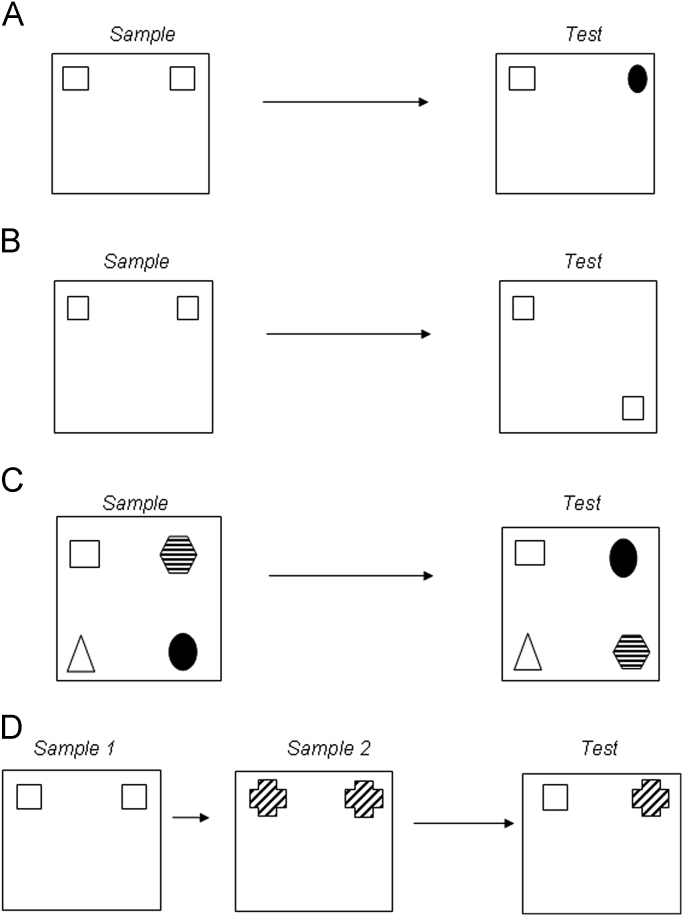
Schematic diagram of four object recognition memory tasks. (A) Novel object preference task (OR), (B) object location task (OL), (C) object-in-place task (OiP) and (D) temporal order task (TO).

The great majority of studies of rodent recognition memory use tasks based on the animals’ spontaneous preference for novelty: a rat (or mouse) will spend longer exploring an object if that object is less familiar or in a less familiar place than an object that is familiar (on the basis of previous exploration) and in a familiar place (Dix & Aggleton, 1999; Ennaceur & Delacour, 1988); see Fig. 1. Such tasks have the advantage of avoiding the potential confounds of using differential rewards or punishments. They have the disadvantage of greater variability and more ready disruption than explicitly reinforced behaviours. Care must be taken if a measure of object familiarity is wanted that is uncontaminated by spatial or associational factors. The most common design for measuring object recognition memory starts with habituating the animal to a display arena. Arenas are typically a square enclosure with four walls that are of the same colour and material and are sufficiently high to mask other features of the room; however, circular arenas, Y-mazes (Winters, Forwood, Cowell, Saksida, & Bussey., 2004), shuttle boxes (Mumby & Pinel, 1994) and ‘bow-tie mazes’ (Albasser, Poirier, & Aggleton, 2010; Albasser, Chapman, et al., 2010) are also used. In a sample or acquisition phase the animal is then allowed to explore (typically for several minutes) two identical copies of an object. After a memory delay (minutes to days), in the choice (or test or retrieval) phase the animal is allowed to explore a third copy of the previously explored object along with a new object occupying the same position as previously occupied by the second copy of the explored object (Fig. 1A). The expectation is that the new object will be explored more than the familiar object. Lesions have established that this task is dependent upon the perirhinal cortex (Barker, Bird, Alexander, & Warburton, 2007; Barker & Warburton, 2011b; Bussey, Muir, & Aggleton, 1999; Ennaceur, Neave, & Aggleton., 1996; Mumby & Pinel, 1994; Norman & Eacott, 2004; Winters et al., 2004).

In the majority of studies, object recognition memory has been found to be unimpaired by hippocampal lesions (Barker & Warburton, 2011b; Forwood, Winters, & Bussey., 2005; Good, Barnes, Staal, McGregor, & Honey, 2007; Langston & Wood, 2010; Mumby, Gaskin, Glenn, Schramek, & Lehmann, 2002; Winters et al., 2004, though see Clark et al., 2000, 2001). It is noteworthy that this hippocampal–perirhinal dissociation is not true for a variant of this task in which two different objects are explored in the sample phase, one of which is replaced in the choice phase, i.e., explore A+B and test A versus C or C versus B, rather than explore A+A and test A versus B (e.g., Clarke, Cammarota, Gruart, Izquierdo, & Delgado-Garcia, 2010; Mansuy, Mayford, Jacob, Kandel, & Bach, 1998; Oh et al., 2010; Pittenger et al., 2002; Barker, G.R.I. unpublished observations). Under the view advanced in this review, in this hippocampally-dependent variant of the object recognition memory task, the rodent also makes spatial or relational associations that require hippocampal processing and which are not made when the sampled objects are both the same; however, that such associations are indeed made remains to be tested experimentally. There are also indications that the involvement of perirhinal cortex and hippocampus may differ if the task employs multiple rather than single exposure trials (Albasser et al., 2011a; Forwood et al., 2005; Gaskin et al., 2010; Mumby, Tremblay, Lecluse, & Lehmann., 2005).

Memory for the familiarity of a location may be tested by starting with a sample phase with two identical copies of an object and, after a delay, measuring exploration of a third copy of the object in the same position compared to that of a fourth copy in a new location (Dix & Aggleton, 1999); see Fig. 1B. The expectation is that the object in the new location will be explored more than the object in the unchanged position. For this task intra- and extra-maze spatial cues are made available to the rat. Lesion studies indicate that this task is dependent on the hippocampus and not perirhinal cortex (Barker & Warburton, 2011b; Mumby et al., 2002), the perirhinal independency presumably reflecting the fact that the task makes no specific demands on object identification.

Memory for the familiarity of a specific association between a particular object and a particular place (object-in-place memory) may be tested by allowing the animal to explore four (or more in some versions) different objects in the sample phase and later testing exploration of two objects that have remained in the same positions against two objects that have exchanged positions (Dix & Aggleton, 1999); see Fig. 1C. Again, intra- and extra- maze spatial cues are made available to the rat. The expectation is that the objects that have moved positions will be explored more than the objects that have stayed in the same place. Lesions have demonstrated that this task is dependent on perirhinal cortex, hippocampus and medial prefrontal cortex (Barker et al., 2007; Barker & Warburton, 2011b; Bussey, Duck, Muir, & Aggleton, 2000).

Recency or temporal order memory may be tested by having two (or potentially more than two) successive sample phases before the choice phase. In the first sample phase two copies of an object are explored and in the second sample phase two copies of a second, different object are explored; see Fig. 1D. Differential exploration of a copy of each of the objects from the first and second sample phases is then measured in the test phase, with the expectation that the object seen first will be explored more than that seen second (Mitchell & Laiacona, 1998). Lesions have demonstrated that this task is dependent on perirhinal cortex, hippocampus and medial prefrontal cortex (Barker et al., 2007; Barker & Warburton, 2011b; Hannesson, Vacca, Howland, & Phillips, 2004a,b; Mitchell & Laiacona, 1998).

### 1.2 Sensory perception and memory

#### 1.2.1 Interference

Perirhinal cortex is at the top end of the ventral visual processing stream (Felleman & Van Essen, 1991) and plays an important role in perceptual as well as memory functions (Bartko, Winters, Cowell, Saksida, & Bussey, 2007a,b; Buckley & Gaffan, 1998, 2006; Bussey & Saksida, 2002; Bussey, Saksida, & Murray, 2002; Murray & Bussey, 1999; Murray et al., 2007; Norman & Eacott, 2004). Accordingly, if memory rather than perceptual deficits are sought it is important that the stimuli used in recognition memory tasks are readily perceptually discriminable. The many studies that have found deficits in recognition memory tasks using two objects with many distinguishable features suggests that the impairment is not readily explained as solely a perceptual failure (Barker et al., 2006a,b, 2007; Seoane, Massey, Keen, Bashir, & Brown, 2009, 2011, 2012; Tinsley et al. 2009, 2011; Tinsley, Narduzzo, Brown, & Warburton, 2012). At the same time, studies in monkeys and rats have established that perirhinal lesions produce perceptual impairment if stimuli have overlapping features so that discrimination requires judgement of differences in stimulus conjunctions within an object and cannot easily be based on single feature differences between objects (Bartko et al., 2007a,b; Buckley, Booth, Rolls, & Gaffan., 2001; Bussey et al., 2002). It has been proposed that recognition memory impairment after perirhinal lesions arises from a greater susceptibility to interference (Bartko, Cowell, Winters, Bussey, & Saksida, 2010; Bussey & Saksida, 2005). Indeed, there is evidence that such an impairment can be removed by preventing interference after acquisition by placing a rat in a dark container (McTighe, Cowell, Winters, Bussey, & Saksida, 2010).

#### 1.2.2 Modalities

Exploration of objects by a rat or mouse in a recognition memory task typically engages more than just the visual system. Even if efforts are made to diminish the importance of olfactory cues, the animal will have available somatosensory information, including the texture and shape of the object as detected by the vibrissae. Regions other than perirhinal cortex are important for recognition memory based on somatosensory information (Albasser et al., 2011a,b; Winters & Reid, 2010). There is also evidence that auditory association cortex is more important than perirhinal cortex for recognition memory for sounds (Fritz et al., 2005; Kowalska et al., 2001; Wan et al., 2001), though perirhinal cortex is necessary for olfactory recognition memory (Otto & Eichenbaum, 1992). However, pharmacological investigations of recognition memory have focused on object exploration and such pharmacological studies have not separately investigated the contributions of different sensory systems.

### 1.3 Pharmacological interventions

As indicated above, ablation studies have established the importance of perirhinal cortex (amongst other regions) for recognition memory. However, a finer dissection of underlying mechanisms is possible by using reversible interventions produced by systemic or localised infusions of compounds with selective actions at particular receptors. Recognition memory is well-suited to pharmacological studies as acquisition can occur in a single brief exposure and the memory formed can last for over 24 h. As in other cortex, perirhinal cortex contains multiple types of receptors for neurotransmitters such as glutamate, γ-amino butyric acid (GABA), acetylcholine and monoamines.

Taking the definition of perirhinal cortex, i.e., areas 35 and 36, from Shi and Cassell (1999), the region is located >4 mm and <6.5 mm posterior to bregma. This definition of perirhinal cortex accords with the region previously investigated in recording studies (Zhu, Brown, & Aggleton, 1995a). It is the region most closely associated with a lesion-induced visual recognition memory deficit (Albasser, Davies, Futter, & Aggleton, 2009), and is differentially activated by novel and familiar individual items in immunohistochemical imaging work (Albasser, Poirier, et al., 2010; Zhu, Brown, McCabe, & Aggleton, 1995b). So defined, perirhinal cortex is sufficiently compact, so that drug infusion via a cannula can be largely confined to it, with there being little spread to surrounding regions. In various regions of the brain where it has been measured, including perirhinal cortex, 1 μl of infusate typically spreads outwards for ∼0.5–1.5 mm from the cannula tip (Attwell, Rahman, & Yeo, 2001; Izquierdo et al., 2000; Martin, 1991).

In many studies the infusate concentration is in the order of 10–100 times the *Ki* value for the target receptor. In spite of a potentially high and necessarily uncertain concentration within the target region, where comparisons have been made results have proved similar to those produced by systemic injections where the concentration delivered within the brain is more readily established. In particular, for metabotropic glutamatergic and cholinergic anatagonists, and the L-type calcium channel blocker verapamil, the same temporal pattern of recognition memory impairment (see further below) has been found when administration is systemic as when it is by infusion into perirhinal cortex. Accordingly, systemic administration that necessarily affects the hippocampus as well as other brain regions in addition to perirhinal cortex does not change the recognition memory deficit found when only perirhinal cortex is targeted. This parallelism of effects between localised perirhinal and systemic administration potentially involving the whole brain strongly argues for the dominant role of perirhinal cortex in the tested recognition memory functions. Moreover, the parallelism of effects between the local perirhinal infusions and systemic administrations indicates that the infusion findings are not distorted by drug concentration gradients that are likely occur with distance from the cannula tip.

Unlike administration by a systemic route, any effects of the compound must result from actions within the perfused region (though it needs to be remembered that these might include compromise of the functioning of distal sites). Thus localised infusion has the advantage of site-specific delivery and the avoidance of potential peripheral side-effects. It is also usable with drugs that do not cross the blood–brain barrier or which would have major detrimental effects on an animal’s health or behaviour if delivered systemically. Notably, here, it is less likely that effects on recognition memory performance will be produced by impairments of global alertness, attention or movement—though the absence of such potential effects should nevertheless be checked through comparisons with the behaviour of controls. Drug effects need always to be compared to the effects of infusion of a similar volume of the vehicle solution, having a matched overall ionic composition, pH and osmolarity.

Importantly, compared with ablation, the effects of infusions are potentially reversible. Though varying with the compound, the actions of many infusates will be established in the target region within 15 min and last for about an hour (e.g., Day, Langston, & Morris, 2003). Infusions may therefore be given so that the infusate is active either during acquisition (and early consolidation), after acquisition and during consolidation, or during retrieval—so allowing potential actions upon memory acquisition, consolidation and retrieval to be separately assessed. However, it needs to be appreciated that at short memory delays drugs given before acquisition are likely to be present also during consolidation and retrieval.

Indeed, drugs active during acquisition may produce recognition memory impairment when retrieval is in the absence of the drug, whereas that impairment disappears if the drug is also active during retrieval. Such an effect is called ‘state-dependency’ as the mnemonic information becomes more readily retrievable when the brain is in the same state as at acquisition. Testing for state-dependency is important when effects of a drug are seen at long but not short memory delays. This is because at a short delay (typically <∼30 min) it is likely that the drug will be active not only during acquisition but also during retrieval, whereas the drug is unlikely to be still active after a longer memory delay (≫1 h).

The results of the large number of pharmacological studies of recognition memory that involve the selective targeting of specific brain regions, almost all performed in the rat, will now be summarised. Most studies have looked at effects on object recognition memory; however, as available, effects of drug delivery in perirhinal cortex upon other types of recognition memory will be contrasted with effects from targeting hippocampus and medial prefrontal cortex. The effects of drugs on acquisition, retrieval and consolidation will be separately considered. Although drugs may affect memory performance by increasing (or reducing) interference between memoranda, they may also impair (or enhance) initial registration or, by acting on consolidation mechanisms, produce (or potentially reduce) temporal decay, or disrupt or enhance retrieval processes.

## 2 Object recognition memory: Drug actions on acquisition

### 2.1 Perirhinal cortex

#### 2.1.1 Glutamate receptors

By far the majority of excitatory neurons in the cerebral cortex use the neurotransmitter glutamate (Collingridge & Lester, 1989) and perirhinal cortex is no exception. There are a large number of different glutamate receptor subtypes, the four main categories being known as AMPA, NMDA, kainate, and metabotropic (see for further details http://www.bristol.ac.uk/synaptic/receptors/). Most fast, excitatory transmission relies on AMPA receptors, with some help from kainate receptors, and NMDA receptors at high transmission frequencies. Metabotropic glutamate receptors act more slowly, but also modulate transmission. Moreover, glutamate receptors are found on both presynaptic and postsynaptic membranes, so allowing feedback autoregulation of transmission.

The compound CNQX antagonises both AMPA and kainate receptors and hence, by stopping almost all excitatory transmission, effectively silences the region perfused. Infused into rat perirhinal cortex, CNQX produces deficits in performance of the spontaneous novel object recognition task (Winters & Bussey, 2005c), thereby reinforcing the findings from ablation experiments. Importantly, infusing CNQX so that it is active during the sample phase, demonstrates that interference with perirhinal processes during acquisition (including early consolidation) can produce recognition memory impairment; the impairment is found without there needing to be further interference with perirhinal processes during retrieval. This is a result that it is not possible to establish with conventional ablation techniques. CNQX produces the impairment by preventing the processing of any perirhinal signal during acquisition and, presumably, also producing some (as yet unknown) disruption of regions targeted by the silenced perirhinal axons. Although important for establishing the necessity of perirhinal activity during acquisition processes, CNQX provides no detailed information concerning which synaptic processes within perirhinal cortex might be those critically involved.

More specific information may be sought using antagonists that target glutamate receptors other than the AMPA receptors necessary for normal fast excitatory synaptic transmission. Compounds that have been shown to impair recognition memory when active in rat perirhinal cortex during acquisition include selective antagonists of NMDA (Barker et al., 2006a; Winters & Bussey, 2005c), kainate (Barker et al., 2006a) and metabotropic (Barker et al., 2006b) glutamate receptors. The impairment when NMDA receptors are antagonised is seen only when the antagonism is of both receptors containing NR2A and those containing NR2B subunits; antagonists selectively targeting either NR2A or NR2B containing NMDA receptors on their own produce no impairment (Barker et al., 2006a). Similarly, it is necessary to antagonise both Group 1 (specifically the mGluR5 subtype) and Group 2 metabotropic glutamate receptors (mGluRs); antagonising either Group 1 or Group 2 receptors alone produces no impairment. Antagonising Group 3 metabotropic glutamate receptors was without effect (Barker et al., 2006b). Impairment of object recognition memory has also been linked to metamphetamine-induced changes in perirhinal mGluR5 receptors (Reichel, Schwendt, McGinty, Olive, & See, 2011). The different time dependencies of the amnesias produced by antagonising the different receptors will be discussed below. The actions of selective antagonism of glutamate receptors establish that recognition memory impairments can be produced without needing to stop all perirhinal synaptic or axonal transmission. Indeed, in perirhinal brain slices maintained in vitro, selective antagonism of NMDA receptors is without effect on normal low frequency synaptic transmission; the effects of such antagonism are upon the induction of synaptic plastic processes (Cho et al., 2000; Ziakopoulos, Tillett, Brown, & Bashir, 1999). This restricted action raises the possibility that impairment remains even when perirhinal cortex is capable of transmission of information to other regions; the implication is that the impairment arises from a failure to store information within perirhinal cortex.

#### 2.1.2 Cholinergic receptors

Neurotoxic lesions of cholinergic neurons made by infusing IgG-saporin into the perirhinal cortex impair object recognition memory at a 15-min delay (Winters & Bussey, 2005a); however, the memory process involved is not discoverable by this non-reversible intervention. Administering antagonists of acetylcholine receptors has established that the recognition memory impairments arise at acquisition. Scopolamine, a broad spectrum muscarinic cholinergic receptor antagonist impairs recognition memory when given by infusion locally into perirhinal cortex before acquisition (Abe & Iwasaki, 2001; Tinsley et al., 2011; Warburton et al., 2003; Winters, Saksida, & Bussey, 2006). Unusually, this impairment is found for shorter (≤3 h) but not longer (6 h, 24 h) memory delays (Tinsley et al., 2011; though see Winters et al., 2006). With systemic injections of scopolamine, recognition memory impairments have been reported with memory delays of 20 min to 3 h (Tinsley et al., 2011), 1 h (Vannucchi et al., 1997) and 3 h (Dodart, Mathis, & Ungerer, 1997) but not 6 h or 24 h (Tinsley et al., 2011). Impairment for a 20 min but not a 24 h delay is also found using perirhinal infusion of pirenzepine, which antagonises M1 receptors, thereby establishing that the memory loss is produced via this subtype of muscarinc receptor (Tinsley et al., 2011). M1 involvement is also demonstrated by the blockade by pirenzepine of improvements in recognition memory that follow administration of an allosteric agonist of M1 receptors (Bradley et al., 2010).

The amnesic actions of scopolamine or interference with cholinergic functioning in monkeys and humans are long established (Aigner & Mishkin, 1986; Aigner, Walker, & Mishkin, 1991; Drachman, 1977; Tang, Mishkin, & Aigner, 1997). Effects on recognition memory in monkeys have not been tested for 24 h delays, and there have been some conflicting results (Browning, Gaffan, Croxson, & Baxter, 2010; Turchi, Saunders, & Mishkin, 2005; Turchi, Buffalari, & Mishkin, 2008). Human studies have concentrated on measuring effects on recall: scopolamine (hyoscine) blocks acquisition rather than retrieval (as for rat recognition memory). However, the amnesia lasts for at least 24 h (e.g., Petersen, 1977), though whether the familiarity discrimination component of recognition memory is only lost for a restricted period of time seems not to have been investigated. Moreover, for humans administration has been systemic, so the site of action is undetermined.

In contrast to the effects of scopolamine in rats, methyllycaconitine (MLA), a nicotinic cholinergic receptor antagonist that includes actions at alpha 7 nicotinic receptors, produces an impairment at delays of 24 h but not 20 min in rats (Tinsley et al., 2011). The same impairment is found whether administration is systemic or by local infusion into perirhinal cortex. These discrepant temporal dependencies of the impairment will be discussed further below. An influence of nicotinic receptors upon recognition memory is also demonstrated by the improved performance that can be produced by systemic administration of nicotinic agonists (Boess et al., 2007; Pichat et al., 2007; Van Kampen et al., 2004). Improvements in recognition memory have also been noted in humans given systemic nicotine, though the effects were state dependent (Warburton, Wesnes, Shergold, & James., 1986). It is of interest that when MLA is administered together with a muscarinic antagonist impairment is not found at all delays: the impairment parallels that produced by MLA unless a very high concentration of scopolamine is used (Tinsley et al., 2011). The temporal pattern of impairment produced seems to depend upon some balance between the antagonism of the two types of receptor; their actions are not additive. It is possible that the complex memory impairments of the differing cholinergic antagonists reflect their effects on the balance of excitation to inhibition in local perirhinal networks, but other explanations cannot be excluded.

#### 2.1.3 Drugs acting on other targets

Effects upon recognition memory of the systemic administration of drugs acting on dopamine, serotonin and other receptors are reviewed elsewhere (Dere et al., 2007; Lyon et al., 2011; Winters et al., 2008).

An acquisition impairment is produced by infusing into perirhinal cortex the benzodiazepine lorazepam (Wan et al., 2004). Benzodiazepines increase the effects of the inhibitory neurotransmitter γ-amino butyric acid at its receptors (Rudolph et al., 1999). Benzodiazepines, including lorazepam, produce recognition memory deficits in humans, the effect also being on acquisition rather than retrieval or faster forgetting (Brown, Lewis, Brown, Horn, & Bowes, 1982; Brown, Brown, & Bowes, 1983; Brown & Brown, 1990; Buffett-Jerrott & Stewart, 2002; Curran, 1991). Unusually, in humans lorazepam (Ativan) also causes impairment in priming memory (Brown, Brown, & Bowes, 1989).

Recognition memory impairments are also produced by L-type voltage-dependent calcium channel blockers (verapamil, diltiazem, nifedipine), given either locally into perirhinal cortex or systemically (Seoane et al., 2009). With either route of administration, deficits are found for a memory delay of 24 h but not 20 min. There is an effect upon acquisition, but also upon retrieval (Seoane et al., 2009). In relation to drug regimes used in human patients, it should be noted that chronically repeated low doses of verapamil have been reported to have, if any, beneficial effects on memory in rats (e.g., Lashgari, Motamedi, Zahedi, Shahidi, & Komaki, 2006; Veng, Mesches, & Browning, 2003)—although the improvements might be indirect, because of improved cardiac performance.

#### 2.1.4 Memory delay dependency of effects

The compounds that interfere with acquisition do not all produce the same pattern of recognition memory impairment: several have effects that are dependent on the length of the delay between acquisition and retrieval. Antagonism of NMDA (Barker et al., 2006a; Winters & Bussey, 2005c) or metabotropic glutamate (Barker et al., 2006b) receptors, or nicotinic cholinergic receptors (Tinsley et al., 2011), or blocking L-type voltage dependent calcium channels (Seoane et al., 2009) produces impairment only for long delays (e.g., 24 h). These antagonists do not impair recognition memory measured after a 5 min or a 20 min delay (Barker et al., 2006a,b; Seoane et al., 2009; Tinsley et al., 2011; Winters & Bussey, 2005c), though Abe, Ishida, and Iwasaki, 2004 found impairment at 25 min using a high dose of the NMDA antagonist AP5. (These effects are not explicable by state-dependency; see further below). For NMDA receptors impairment is produced for memory delays ≥1 h (Barker & Warburton, 2008a,b).

In contrast, antagonism of kainate glutamatergic (Barker et al., 2006a) or muscarinic cholinergic (Tinsley et al., 2011) receptors produces impairment at short (≤20 min) but not long delays (>1 h for kainate (Ho, *unpublished observation*) and >3 h for muscarinic antagonism). Strikingly, in this unusual pattern of impairment, amnesia is followed by remembrance. The double dissociation of the time courses of amnesic effects for both glutamatergic and cholinergic receptor antagonism indicates that there must be more than one underlying mechanism supporting recognition memory. Moreover, both these recognition memory mechanisms are dependent upon the intact operation of perirhinal cortex. The possibility that a region other than perirhinal cortex is sufficient to support the memory at short (20 min) or at long (24 h) delays may be excluded. First, perirhinal lesions not only produce recognition memory deficits at long delays but do so even at very short delays (<1 min) (Aggleton, Albasser, Aggleton, Poirier, & Pearce, 2010; Albasser, Poirier, et al., 2010; Albasser, Chapman, et al., 2010). Second, if the effect of NMDA receptor antagonism within perirhinal cortex at short delays was masked by some other region, the effect of kainate receptor antagonism within perirhinal cortex at such short delays should also be masked, but it is not. Reversing the argument for kainate antagonism at long delays, the effect of NMDA antagonism within rat perirhinal cortex fails to be explained. Memory at both long and short delays is dependent upon the unimpaired operation of perirhinal cortex.

**Fig. 2:**
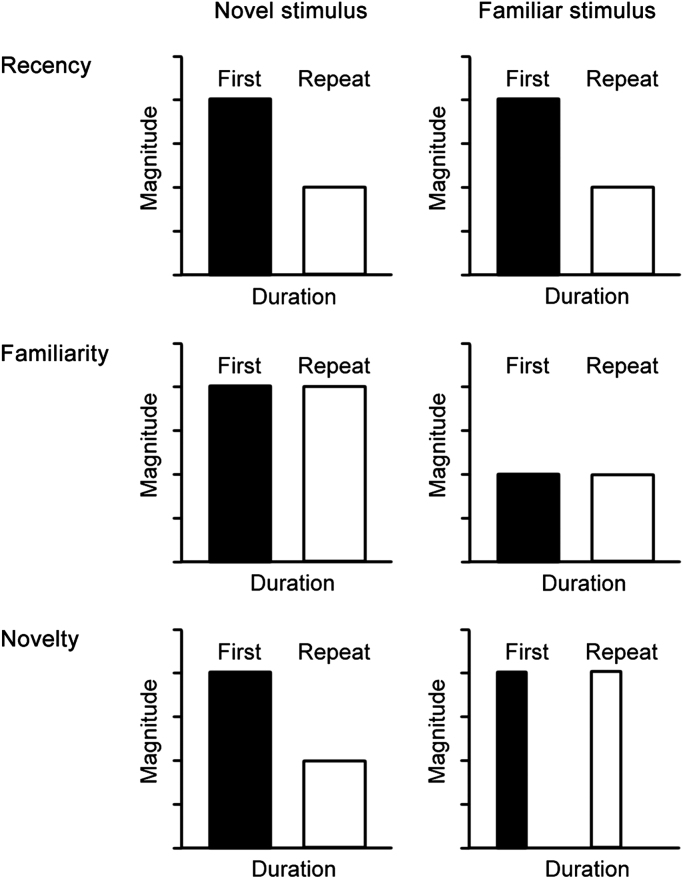
Schematic representation of different patterns of response reduction on stimulus repetition. There are three patterns of neuronal response reduction found on repetition of novel and highly familiar visual stimuli found in monkey anterior inferior temporal cortex, including the perirhinal cortex. Neurons with ‘recency responses’ signal that a stimulus has been seen recently by a reduced response to that stimulus, but do not signal whether it is unfamiliar or highly familiar, because the response to both types of stimulus is the same. ‘Familiarity responses’ signal that a stimulus is highly familiar (has been seen many times on previous days) by a reduced response to such a stimulus but do not signal that a stimulus has been seen recently (within the past several minutes), because the responses to its first and second occurrence are the same. ‘Novelty responses’ signal that the stimulus is being seen for the first time by a vigorous response that is much weaker when the stimulus is repeated and much briefer (thinner bar) if the stimulus is highly familiar. (After Fig. 17 of Brown & Xiang (1998); reproduced with permission).

**Fig. 3:**
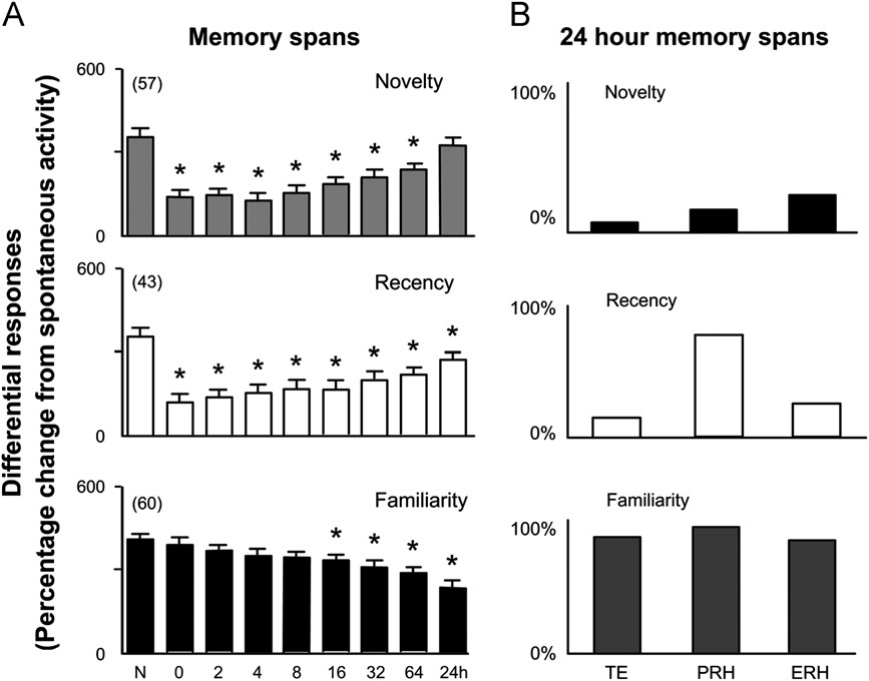
Neuronal memory spans. (A) Responses averaged across populations of novelty, recency and familiarity neurons recorded in anterior inferior temporal cortex in the monkey. The mean response (% of background activity) to novel stimuli is shown in by the first bar (‘N’). Subsequent bars give the mean response to repeat presentations after the indicated number of intervening trials (0–64) or after a 24 h delay. Note that for novelty and recency neurons the response to repeat presentations is reduced and that the magnitude of the reduction decreases as the intervening interval increases (‘forgetting curve’). In contrast, for familiarity neurons there is no significant reduction at short intervals but then the reduction increases with interval. ^⁎^ Difference from response to novel, *p*<0.05. (B) The proportion of neurons with reduced response on stimulus repetition where the reduction was still significant after a 24 h interval between initial and subsequent presentation. Data are shown for novelty, recency and familiarity neurons in anterior visual association cortex (TE), perirhinal cortex (PRH) and entorhinal cortex (ENT). Note the high proportion of familiarity neurons with memory spans of ≥24 h. Data from Xiang & Brown (1998).

Such a possible double dissociation has not been pharmacologically investigated in the monkey. However, single neuronal recordings in the monkey had already indicated the requirement for more than one underlying synaptic plastic process (Brown & Xiang, 1998; Fahy, Riches, & Brown, 1993; Xiang & Brown, 1998). Many neurons in perirhinal and adjacent medial temporal lobe cortex in the monkey respond less strongly to stimuli that have been seen before than they do to unfamiliar stimuli (see for review, Brown & Xiang, 1998). However, when response changes are compared for first and repeat presentations both of novel stimuli and of highly familiar stimuli, three different patterns are found (see Fig. 2). It is not possible to produce these different, doubly dissociable patterns of responsiveness if there is only one underlying synaptic plastic process (Brown & Xiang, 1998; Fahy et al., 1993). In Fig. 3A changes in responsiveness of monkey neurons upon stimulus repetition are plotted against memory delay (number of trials intervening between first and repeat presentations). Certain neurons (‘novelty’ and ‘recency’) show reduced responses even if stimuli are re-presented after a very short delay (in other experiments <1 s (Miller, Li, & Desimone, 1993). The response changes of these neurons require a fast acting synaptic plastic change. In contrast, certain other neurons (‘familiarity’) only show reduced responses when some time (several minutes) has elapsed. The response changes of these neurons require a slowly developing synaptic plastic change. It has been suggested that the reason for having more than one pattern of response change is to make possible the separate assessment of familiarity and recency of occurrence (Brown & Xiang, 1998; Fahy et al., 1993).

**Fig. 4:**
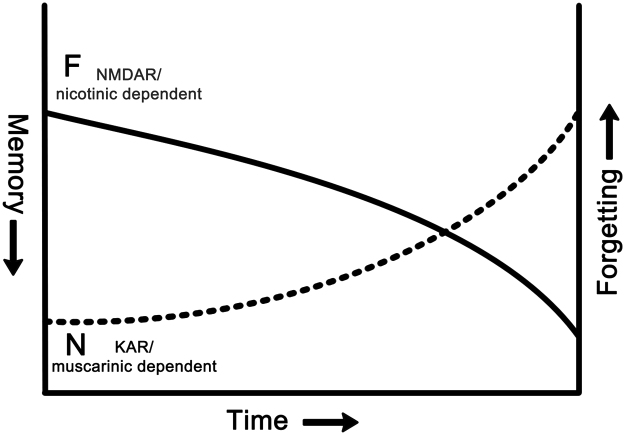
Differential memory decays (forgetting curves) produced by selective glutamatergic and cholinergic antagonists. The curves follow the ‘forgetting curves’ of the different neuronal types – novelty and recency (N) compared to familiarity (F) – as shown in Fig. 3A. If kainate (KAR) and muscarinic receptor antagonists target ‘novelty’ and ‘recency’ (fast synaptic change) neurons, while NMDA and nicotinic receptor antagonists target ‘familiarity’ (slow synaptic change) neurons, then the different forgetting curves provide potential explanation for the different amnesic effects observed: NMDA or muscarinic antagonism results in short-term memory followed by forgetting at longer intervals, whereas kainate (KAR) or muscarinic antagonism produces short-term forgetting followed by long-term remembrance.

Given these two types of response change, evidence of memory for a prior occurrence at short time delays is not signalled by responses of the slowly changing neurons, but it is signalled by the fast changing neurons. Plotted in Fig. 2B are the incidences of neurons whose activity signals prior occurrence even after a 24 h delay. It is clear that the proportion of slowly changing neurons (‘familiarity’) is higher than that for the fast changing neurons (‘novelty’ and ‘recency’). Accordingly, after such long delays evidence for prior occurrence is more strongly signalled by the slowly changing neurons, so that memory is better based on their activity than on that of the fast changing neurons. The corresponding data for the rat are not known—though both types of response changes have been observed in rat perirhinal cortex (Zhu et al., 1995a), how the changes are maintained across time has not been established for the rat. However, should the pattern of response change across time for the two classes of neurons be similar in the rat to the monkey, then the pharmacological double dissociations can be explained (Barker et al., 2006a; Tinsley et al., 2011). If kainate or muscarinic receptor antagonism blocks the response change only in fast synaptic change neurons, then memory at short intervals will be impaired because the memory cannot be based upon the responses of the slow synaptic change neurons (Fig. 4). In contrast, at long delays the memory for prior occurrence can be based on the response changes of the unaffected slow synaptic change neurons. Correspondingly, if NMDA or nicotinic receptor antagonism blocks the response change only in slow synaptic change neurons, then memory at long but not short intervals will be impaired (Fig. 4). This follows because the memory at short intervals can be based on the unaffected fast change neurons, while that at long delays cannot be based upon the responses of the slow synaptic change neurons.

The double dissociation of amnesic effects at short and long delays produced by these glutamatergic and cholinergic antagonists provides evidence against increased susceptibility to interference (Bartko et al., 2007a,b, 2010; McTighe et al., 2010; Winters, Bartko, Saksida, & Bussey, 2007) as the sole ground for drug-induced loss of recognition memory. If increased interference with the passage of time after acquisition is invoked to explain the memory loss produced at longer delays by NMDA and nicotinic antagonists, the recovery of memory at longer delay after administration of kainate or muscarinic antagonists remains unexplained. The alternative explanation given above suggests the differential amnesias arise through impairments of synaptic plasticity mechanisms with different time courses of expression.

The presence of at least two underlying memory processes complicates interpretation of the effects of drugs on perirhinal-dependent recognition memory. Importantly, the effects of a drug need to be measured at both long (>3 h) and short (<1 h) delays if the full extent of its actions are to be understood. Lack of amnesic effect at one delay (e.g., short) does not predict the effect at another (e.g., long). Moreover, effects on acquisition, consolidation or retrieval mechanisms may be misinterpreted, notably if an effect at short delay is masked by a parallel, unaffected process. Clearly, more than one process for the induction of synaptic plasticity needs to be sought. As mentioned above, the time-dependency of the effects of scopolamine upon recognition memory in humans remains to be investigated, and kainate antagonists are not licensed for human use.

### 2.2 Other areas

To investigate the extent to which perirhinal function may be dependent upon or duplicated by other brain regions, studies of the effects upon recognition memory acquisition produced by pharmacological infusions to other areas will now be considered. Other parts of the visual system are likely to provide input and may receive output from the perirhinal cortex. The hippocampal system continues to be of interest as a potential partner of perirhinal cortex in recognition memory functions. There have, however, been relatively few such studies.

#### 2.2.1 Visual association cortex

##### 2.2.1.1 Area Te2

The region of association cortex dorsally adjacent to perirhinal cortex, here termed Te2 (Sia & Bourne, 2008; Shi & Cassell, 1997), is strongly interconnected with perirhinal cortex, in particular providing it with visual information (Burwell & Amaral, 1998; Sia & Bourne, 2008; Shi & Cassell, 1997). Neuronal responses and immunohistochemical changes related to recognition memory have been reported there (Tinsley et al., 2009; Wan, Aggleton, & Brown, 1999; Zhu et al., 1995a,b). In this respect it has parallels with area TE in the monkey (Brown & Xiang, 1998).

Infusions of antagonists into Te2 produce object recognition memory deficits at long (24 h) but not short (20 min) delays (Ho et al., 2011). Thus infusions of selective antagonists of AMPA and kainate (CNQX) or NMDA (AP5) or kainate glutamate receptors each produce this pattern of amnesia (Ho et al., 2011). The finding for NMDA antagonism parallels that for perirhinal cortex (Barker et al., 2006a; Lopez-Aranda et al., 2009; Winters & Bussey, 2005c). However, CNQX fails to produce an impairment for a 20 min memory delay showing that a functioning Te2 during acquisition, consolidation and retrieval is not necessary for recognition memory at this delay. Hence some other region (presumably perirhinal cortex) must be responsible for recognition memory at a delay of 20 min. Infusion of the muscarinic cholinergic antagonist scopolamine into Te2 fails to produce an impairment at either a 20 min or 24 h delay (Ho et al., 2011). If the action of scopolamine in Te2 is similar to that in perirhinal cortex (Tinsley et al., 2011), any resulting impairment would be limited to memory delays of ≤3 h. Hence any possible effect upon Te2 at 20 min would be masked because Te2 is not necessary for the memory at that delay. No effect would be predicted for memory delays of 24 h, as found for Te2 infusions of the drug. The action of kainate antagonism contrasts with that in perirhinal cortex. In perirhinal cortex recognition memory impairment is found for a 20 min but not a 24 h delay (Barker et al., 2006a). In Te2, for the same reason as given above, whether kainate antagonism produces an impairment of Te2 functioning at a 20 min delay is unknown; however, there is a recognition memory impairment for a 24 h delay with infusions into Te2 but not perirhinal cortex.

Thus there are differences in the synaptic mechanisms underlying recognition memory processes in perirhinal cortex and area Te2. It may also be concluded that for object recognition memory measured at a 24 h delay normal functioning is required for both perirhinal cortex and Te2. In contrast, at short delays (≤20 min) a normally functioning perirhinal cortex is essential, but Te2 is not required.

Visual information is fed forward from area Te2 to PRH (Burwell & Amaral, 1998; Sia & Bourne, 2008; Shi & Cassell, 1999), though there is also direct input to PRH from occipital visual areas (Burwell & Amaral, 1998). Hence interference with processing in Te2 may result in impairment of recognition memory through the interruption of the transfer of visual information to perirhinal cortex. Although impairment from such interruption is to be expected, this does not provide a complete explanation of the amnesia as perception (as measured by the simultaneous discrimination of objects such as used in the memory tasks) and short-term recognition memory were not impaired (Ho et al., 2011).

It is perhaps unexpected that the dependency should be that Te2 is required for longer rather than shorter-term object recognition memory. One possibility is that a deficit is only seen at longer delay because at longer delay the task is more difficult, in particular being liable to greater interference exacerbated by a degradation of visual perceptual processing. Another possibility is that the dependency upon Te2 at longer delays arises from signals fed back from perirhinal cortex to Te2. Structural equation modelling based on Fos imaging has indicated the presence of such feedback (see Aggleton et al., this issue; Albasser, Poirier, et al., 2010). Additionally, there is evidence of backwardly propagating signals from perirhinal cortex for visual paired associate learning in the monkey (Naya, Yoshida, & Miyashita 2001, 2003). Localised inhibition of either the phosphorylation of calcium–calmodulin dependent protein kinase II (CAMKII) or calcium–calmodulin dependent protein kinase kinase (CAMKK) in perirhinal cortex results in loss of the differential phosphorylation of CAMKII or CAMKK by novel and familiar stimuli in Te2, i.e., for CAMKII and CAMKK the change in Te2 is dependent on PRH activity (Tinsley et al., 2009, 2012). In contrast, differential Fos expression in Te2 is not dependent on the unimpaired operation of Fos-dependent consolidation processes in perirhinal cortex (Seoane et al., 2012). If these activity differences in Te2 are related to a signal that passes from perirhinal cortex to Te2 the results suggest that this signal must pass after the time that CAMKII has been activated, i.e., >20 min after acquisition (Tinsley et al., 2009, 2012), but before further processing has been disrupted by preventing Fos expression, i.e., <3 h after acquisition (Seoane et al., 2012). Even should there prove to be such a backwardly propagating signal, activity in Te2 is insufficient to maintain recognition memory after a 24 h delay if perirhinal processing is disrupted at the time of retrieval (see below for effects of perirhinal disruption during retrieval).

##### 2.2.1.2 Visual cortex area *V*2

There is a single report of viral transduction resulting in the overexpression of RGS-14 protein (a protein involved in G protein signalling) in layer 6 of visual cortex area *V*2 improving object recognition memory performance (López-Aranda et al., 2009). No effect was seen for such transduction within hippocampus; effects in perirhinal cortex were not sought. No current theory would predict that interfering with such an early stage of the visual processing pathway would produce recognition memory enhancement.

#### 2.2.2 Nucleus accumbens

For completeness and because the nucleus accumbens receives hippocampal outputs, two sets of experiments concerning recognition memory will be mentioned here. The nucleus accumbens is implicated in reward mechanisms and receives direct hippocampal inputs as well as dopaminergic fibres from the ventral tegmental area. Infusions of NMDA (AP5) or AMPA (DNQX) glutamate receptor antagonists into the nucleus accumbens have been reported to produce object (Sargolini, Roullet, Oliverio, & Mele, 2003) and object-in-place (Roullet, Sargolini, Oliverio, & Mele, 2001) recognition memory impairments in mice when infused before (Object: AMPA & NMDA; Object-in-Place: AMPA) or after acquisition (Object & Object-in-Place: NMDA) or before retrieval (Object-in-Place: AMPA). The involvement of the nucleus accumbens with reward mechanisms raises the possibility that the reported impairments in the preferential exploration of novelty may arise from alterations in novelty seeking behaviour rather than in memory per se.

#### 2.2.3 Hippocampus

Most studies involving hippocampal interventions have been concerned with consolidation mechanisms and are discussed in the corresponding section below. However, inactivation produced by lidocaine has been reported to produce impairment in object recognition memory in mice when infused into the hippocampus before acquisition with memory measured after a 24 h but not a 5 min delay (Hammond, Tull, & Stackman, 2004). Additionally, there is a report that intrahippocampal infusion of the NMDA antagonist AP5 before acquisition impaired object recognition memory in rats when tested after a delay of 3 h, but not 5 min (Baker & Kim, 2002). As may be seen, the number of such reported impairments with hippocampal infusions is small compared to the corresponding list for perirhinal cortex.

#### 2.2.4 Septal nuclei

The septal nuclei are strongly interconnected with the hippocampus. Intra-septal infusion of an NMDA antagonist (AP5) improved object recognition memory measured after a 24 h delay, but had no effect with a 45 min delay, whether infusion was before or after acquisition or before retrieval (Puma & Bizot, 1998). It is possible that the improvement is a result of some induced bias towards enhanced perirhinal rather than hippocampal processing when there is interference with septal and, hence, with hippoocampal functioning.

#### 2.2.5 Prefrontal cortex

Infusion before acquisition of a dopamine (D1 receptor) antagonist into prefrontal cortex in mice produced an impairment in the hippocampally-dependent version of the object recognition memory task when tested after a 24 h but not a 1 h delay (Nagai et al., 2007). In general, as reviewed later, prefrontal interference affects object-in-place and temporal order tasks rather than object recognition memory. As a dopamine antagonist was used, the impairment here may arise from effects on the reward/behavioural drive mechanisms rather than on memory per se.

In summary of Section 2.2, there has been relatively little work on the dependency of recognition memory acquisition on areas outside perirhinal cortex. Even so, such work as there has been fails to challenge the central role of perirhinal cortex in recognition memory acquisition for object occurrence.

## 3 Object recognition memory: Drug actions on retrieval

It is an unfortunately commonplace human experience that memory retrieval sometimes fails (i.e., a memory that fails to be retrieved at one time is successfully retrieved at a later time). Indeed, in the past, retrieval failure (as a result of faster forgetting) has been advanced as an explanation of anterograde amnesia (e.g., Weiskrantz & Warrington, 1970). However, it is less common for such failure to be related to object familiarity than to recollective aspects of recognition memory. As reviewed below, pharmacological studies of object recognition memory in rats suggest that disruption of perirhinal cortex during retrieval less frequently impairs memory than does the same disruption during acquisition. The implication is that perirhinal processes for laying down new information are more readily disturbed than those for retrieving previously learned material.

### 3.1 Perirhinal cortex

In contrast to the impairments found when drugs are infused into perirhinal cortex so as to be active during acquisition, most compounds that have been tested do not have any effect on rat recognition memory when infused so as to be active during retrieval. This is not because perirhinal cortex is unnecessary for recognition memory retrieval processes. CNQX infusion during the choice phase produces recognition memory impairment (Winters & Bussey, 2005c). This result establishes that perirhinal cortex is necessary for object recognition memory during the retrieval process as well as during acquisition, again a result that it is not possible to establish with conventional ablation techniques. This impairment arises because CNQX shuts down perirhinal cortex activity during retrieval, so preventing the processing of any perirhinal signal at that time. Confirmation of the involvement of perirhinal cortex in retrieval processes is provided by the impairment produced by local infusion of the sodium channel blocker lidocaine (Barnes, Floresco, Kornecook, & Pinel, 2000; Hannesson et al., 2004b; Winters & Bussey, 2005b). It should, however, be noted that since lidocaine blocks axonal transmission, its infusion will also involve fibres that merely pass through perirhinal cortex rather than terminating or originating there.

CNQX and lidocaine (Hanneson et al., 2004a,b; Winters & Bussey, 2005b,c) impair both acquisition and retrieval, but most drugs that have been tested impair acquisition but not retrieval. An exception is provided by the blockade of voltage-dependent calcium channels by verapamil: blockade of such channels at either acquisition or retrieval produces recognition memory deficits (Seoane et al., 2009). A disjunction between acquisition impairment and a lack of effect on retrieval has been demonstrated for antagonism of NMDA (Barker et al., 2006a; Winters & Bussey, 2005b, though see Abe et al., 2004), kainate (Barker et al., 2006a), and metabotropic glutamate receptors (Barker et al., 2006b), and muscarinic cholinergic receptors (Tinsley et al., 2011; Warburton et al., 2003; Winters, Saksida, & Bussey, 2006, though see Abe et al., 2004 and, using mice, Botton et al., 2010), and for the benzodiazepine lorazepam (Wan et al., 2004). These findings strongly indicate that it is easier to disrupt recognition memory by interfering with perirhinal processes during acquisition than when the same interference occurs during retrieval. Accordingly, either acquisition processes are more readily disrupted than retrieval processes or perirhinal cortex is in some way less critical for retrieval than for acquisition. The former is more likely to be the case. First, in other brain regions and for various tasks acquisition is also found to be more readily disrupted than retrieval (see for review, Squire, 2006). Second, the impairments produced by CNQX and verapamil establish the involvement of perirhinal cortex in retrieval processes (Seoane et al., 2009; Winters & Bussey, 2005c): the lack of effects of other drugs is not because some other region has rendered perirhinal activity unnecessary. Indeed, the disruptive effects of the drugs upon acquisition rather than retrieval are most easily explained by their interference with the conditions necessary for synaptic plasticity to occur, with the consequent impairment of the laying down of the memory.

It is worth noting that there has not been determination of the maximum length of time for which information concerning a specific example of prior occurrence is held in perirhinal cortex. For memories that depend upon the hippocampus at acquisition, there is accumulating evidence that at least some of them become independent of the hippocampus over a period of days to weeks (see for recent review, Winocur & Moscovitch, 2011). However, the potential storage capacity of perirhinal cortex for familiarity discrimination of objects is sufficiently high that such storage might reside permanently within the perirhinal cortex (Androulidakis et al., 2008; Bogacz & Brown, 2003).

### 3.2 State dependency

Where investigated in studies of localised drug action within perirhinal cortex, no evidence of state-dependent effects has been found for rodent recognition memory. In particular, neither NMDA (Barker et al., 2006a) nor metabotropic glutamate receptor (Barker et al., 2006b) antagonism demonstrated state-dependency. Therefore for these antagonists the lack of impairment at shorter delay (15–20 min) compared to the impairment at longer (24 h) delay is not explained by state-dependency. However, a state-dependent recognition memory impairment has been reported for infusions of a nitric oxide synthesis inhibitor (nomega-nitro-l-arginine) into the hippocampus (Blokland, Prickaerts, Honig, & de Vente, 1998).

In human studies state dependency has been reported for the cholinergic muscarinic antagonist scopolamine (Petersen, 1979) and cholinergic agonist nicotine (Warburton et al., 1986).

## 4 Object recognition memory: Drug actions on consolidation

Memory loss, forgetting, in intact humans is ascribed to interference and/or temporal decay. As already discussed, interference has been advocated as an explanation for forgetting within recognition memory on the basis of the results of animal experiments (Bartko et al., 2010; Bussey & Saksida, 2005). Indeed, computational models of recognition memory demonstrate how such interference (overwriting of synaptic weight changes) could give rise to reduced performance, i.e., forgetting, or the generation of false positives (Bartko et al., 2010; Lulham, Bogacz, Vogt, & Brown, 2011; Norman and O'Reilly, 2003). However, failures of consolidation or maintenance will also produce forgetting, effectively by temporal decay (the material is not adequately maintained across time). As the following section will demonstrate there is much animal evidence that such ‘temporal decay’ can be readily produced. In general, the interventions employ drugs that cannot ethically be used in humans. Correspondingly, there is a lack of parallel human data, though this highlights the value of the animal studies to the development of a full understanding of the brain processes underlying recognition memory.

Any drug that is active during the sample phase of a recognition memory task may affect early consolidation as well as acquisition processes (though there is probably a continuum rather than a clear dividing line between brain processes involved in acquisition and consolidation). However, consolidation processes may be targeted independently of acquisition by infusing a drug after the sample phase (discounting the possibility that an animal might continue to rehearse what it has experienced during the sample phase). Consolidation of information into memory is a complex cascade of processes that continues over minutes to hours or even, in the case of systems consolidation, weeks (see for review, Dudai & Morris, 2000). Long-term consolidation involves protein synthesis (Davis & Squire, 1984; Kandel, 2001). Indeed, inhibition of such synthesis with infusions of anisomycin into perirhinal cortex impairs object recognition memory measured after a 24 h but not a 90 min delay (Balderas et al., 2008).

There is increasing evidence that sleep affects consolidation (see for review, Walker & Stickgold, 2006), a factor that so far has received little discussion in behavioural studies of the neural basis of recognition memory. Potentially, therefore, there may be differences in drug effects on recognition memory depending on whether or not animals have slept during the delay before test. Sleep may allow the activation of different consolidation mechanisms—but this issue remains to be addressed in relation to recognition memory. A further topic that has as yet received little investigation in relation to recognition memory is memory ‘reconsolidation’ (Nader, Schafe, & LeDoux, 2000). There is now substantial evidence that previously established memories become newly labile when they are retrieved or reactivated by a closely similar experience and consequently need to undergo some form of repeat consolidation i.e., ‘reconsolidation’ (Dudai, 2012; Nadel, Hupbach, Gomez, & Newman-Smith, 2012; Nader et al., 2000). At such a time the memory may be disrupted. Drug effects on such reconsolidation mechanisms within perirhinal cortex remain to be systematically investigated, though impairment of recognition memory attributed to blocking reconsolidation mechanisms has been recently reported to follow inhibition of perirhinal protein synthesis (Romero-Granados, Fontan-Lozano, Delgado-Garcia, & Carrion, 2010). In contrast, when scopolamine was infused into perirhinal cortex before presentation of an additional object between initial acquisition and subsequent retrieval phases of an object recognition memory task, memory for the object shown at acquisition was unimpaired (Winters et al., 2007). Invoking the hypothesis underlying reconsolidation, showing the additional object might have been expected to render the original memory labile and thereby vulnerable to the amnesic actions of scopolamine. However, it is possible that muscarinic receptor antagonism by scopolamine, as opposed to protein synthesis inhibition, fails to target the appropriate reconsolidation mechanisms.

### 4.1 Perirhinal cortex

Recognition memory impairments are found when CNQX or lidocaine is infused into perirhinal cortex very shortly after the sample phase so as to be active during early consolidation (Winters & Bussey, 2005b,c). No impairment is found if infusion is >40 min after acquisition (Winters & Bussey, 2005b,c). Accordingly, shutting down perirhinal activity shortly after acquisition causes impairment. This suggests either that activity-dependent processing (extracellular signalling) continuing in perirhinal cortex during the first hour after acquisition is necessary for consolidation, or that silencing the neurons during this time causes disruption of intracellular signalling, thereby interfering with normal consolidation. No tests have been made of the effects of infusions preventing perirhinal activity over many hours.

The effect of post-acquisition intra-perirhinal infusion of an NMDA receptor antagonist (AP5) on consolidation is equivocal as impairment was not seen in one study (Barker et al., 2006a) but was in another (Winters & Bussey, 2005c). Impairment was also found when AP5 was present during both consolidation and retrieval when the memory delay was 25 min (Abe et al., 2004). It should be noted that any impairment with post-acquisition infusion undermines the argument that NMDA receptor antagonism produces impairment by acting solely at acquisition. However, studies in perirhinal cortical slices maintained in vitro indicate AP5 blocks the induction of artificially produced synaptic plasticity rather than its maintenance (Ziakopoulos et al., 1999), so the predicted impairment is of acquisition. Kainate and metabotropic glutamate receptor antagonism was without effect on consolidation (Barker et al., 2006a,b). Again, metabotropic glutamate receptor activation has been shown to be important for perirhinal plasticity mechanisms (Cho et al., 2000).

Post-acquisition intra-perirhinal infusion of the muscarinic receptor antagonist scopolamine results in improved performance (Winters et al., 2006). This improvement was interpreted as arising from decreased interference (Winters et al., 2006), as has been reported for alcohol, nitrous oxide, and benzodiazepine -induced amnesias in human subjects (Brown et al., 1982; Parker et al. 1980; Summerfield & Steinberg, 1957).

The current leading model of memory formation presumes that activity-dependent release of neurotransmitters and their consequent binding to their receptors leads to changes in intracellular concentrations of calcium ions that in turn trigger biochemical cascades that lead to synaptic plasticity (e.g., Bading, Ginty, & Greenberg, 1993; Kotaleski & Blackwell, 2010; Citri & Malenka, 2008), i.e., they result in changes in synaptic efficacy and the maintenance of that change. Indeed, there is a report of synaptic remodelling in perirhinal cortex after object recognition memory training (Platano et al., 2006). One established mechanism of changing synaptic efficacy is to alter the number of AMPA glutamate receptors at the synapse (Collingridge, Isaac, & Wang, 2004; Nicoll and Malenka, 1999); increasing the number will produce strengthening (as for example seen in long-term potentiation); reducing it will produce weakening (as for example seen in long-term depression). By hypothesis, the memory trace is represented by the maintained changes in synaptic efficacy across the whole of the network involved (see for discussion, Martin, Grimwood, & Morris, 2000).

Infusing into perirhinal cortex drugs that target intracellular processes related to synaptic plasticity mechanisms (potential consolidation mechanisms) has been shown to produce recognition memory impairments. Most such investigations have concerned object recognition memory. Typically effects have been seen on memory measured after a 24 h but not a 20 min delay. The lack of effect on memory after a 20 min delay may be because either the consolidation mechanisms being studied are unnecessary for memory at such a short delay or that the underlying synaptic plasticity mechanisms differ (as discussed above).

Calcium–calmodulin-dependent kinases (CamKs) are enzymes that are activated as a result of increases in intracellular calcium ions. In turn, these kinases phosphorylate other enzymes involved in intracellular signalling processes necessary for memory formation, as has been shown for other types of memory dependent on the hippocampus or amygdala (e.g., Giese, Fedorov, Filipkowski, & Silva, 1998; Lisman, Schulman, & Cline, 2002; Rodrigues, Farb, Bauer, LeDoux, & Schafe, 2004; Wayman et al., 2008). Recognition memory impairments are produced by infusing into perirhinal cortex inhibitors of these enzymes so as to interfere with consolidation mechanisms. Infusion of an inhibitor of CamK kinase (CamKK) immediately post-acquisition but not 20 min after acquisition impaired object recognition memory (Tinsley et al., 2012). Thus CamKK is important for perirhinal consolidation mechanisms necessary for recognition memory within the first 20–30 min after acquisition. CamKK phosphorylates CamKI. As expected, the CamKK inhibitor also reduced the phosphorylation of CamKI; however, CamKI phosphorylation was similarly reduced in response to the viewing both of novel and of familiar stimuli. This suggests that the effect on CamKK is not selective for the registration of the occurrence of the novel stimulus (Tinsley et al., 2012). The effect of the inhibitor may thus be a generalised downgrading of cellular function or it might act on reconsolidation (of the occurrence of the familiar object) as well as consolidation (of the occurrence of the novel object) mechanisms. Inhibition of CamKII within perirhinal cortex 20–100 min after acquisition impairs object recognition memory (Tinsley et al., 2009). Such inhibition of CamKII prevented the increased phosphorylation of CamKII produced by viewing novel rather than familiar images, suggesting that phosphorylation of CamKII is selectively related to the processing of information concerning novel stimuli. These experiments also indicated that CamKIV did not compensate for the inhibited CamKII phosphorylation in long-term object recognition memory (Tinsley et al., 2009). Thus the evidence suggests that CamKII phosphorylation is more closely related to the registration of novel information in perirhinal cortex than either CamKI or CamKIV.

One of the targets of phosphorylated CamKII is calcium response element binding protein (CREB). CamKII activates CREB by phosphorylating it (forming pCREB). pCREB then becomes active by dimerising. pCREB can be prevented from dimerising by using viral transfection to produce a dominant negative construct that binds pCREB. When this is done in perirhinal cortex, long-term recognition memory is impaired (Warburton et al., 2005). As viral transfection rather than an infusion was used, the time after acquisition at which consolidation might be disrupted could not be established (indeed, that the effect is on consolidation must be surmised).

In turn, one of the eventual targets affected by CREB phosphorylation is the immediate early gene c-*fos*. Changes in Fos, the protein products of c-*fos*, have provided a reliable marker for changes in neuronal activity related to recognition memory across many studies (see for review, Aggleton et al., this issue). Fos has been implicated in synaptic plastic mechanisms that produce reductions in efficacy (Lindecke et al., 2006; Nakazawa, Karachot, Nakabeppu, & Yamamori, 1993). Reductions in synaptic strength have been predicted to underlie familiarity discrimination (Brown & Aggleton, 2001; Brown & Xiang, 1998). Infusion into perirhinal cortex of an oligodeoxynucleotide (ODN) that prevents the production of Fos also produces an impairment in long-term (but not shorter-term) recognition memory (Seoane et al., 2012). A similar impairment was produced by perirhinal infusion of an ODN against brain-derived neurotrophic factor (BDNF). Additionally, expression of BDNF has been reported to be increased in perirhinal cortex after performance of an object recognition memory task (Griffin, Bechara, Birch, & Kelly, 2009; Hopkins, Nitecki, & Bucci, 2011; Muñoz, Aspé, Contreras & Palacios, 2010).

As mentioned above, it has been proposed that the primary plastic change underlying object recognition memory is synaptic weakening (Brown & Aggleton, 2001; Bogacz & Brown, 2003; Brown & Xiang, 1998). Such weakening would explain why responses to a new stimulus are reduced when that stimulus is seen again, as is found in perirhinal and neighbouring cortex (Brown & Xiang, 1998). Indeed, computational modelling has established that efficient familiarity discrimination must employ some element of synaptic weakening (Bogacz & Brown, 2003; Lulham et al., 2011). Activity-dependent removal of AMPA glutamate receptors from the synaptic membrane is the most obvious (but not the only) way in which to reduce excitatory synaptic strength. Such removal can be prevented by blocking the appropriate intracellular machinery—specifically by preventing AP2 (clathrin adaptor protein 2) from binding to the GluR2 subcomponent of AMPA receptors, a necessary step for the receptor’s removal from the membrane (Androulidakis, Lulham, Bogacz, & Brown, 2008). This impairment across the memory delays indicates that although there may be more than one method for inducing synaptic change in perirhinal cortex, these methods are dependent upon the same expression mechanism, namely internalisation of AMPA receptors.

Interestingly, this prevention of AMPA internalisation did not impair object-in-place memory (though this was only tested at a short memory delay). This lack of impairment indicates that object-in-place recognition memory does not share the same underlying synaptic plastic change with object recognition memory in perirhinal cortex. Preventing the activity-dependent removal of AMPA receptors by blocking AP2’s interaction leaves normal synaptic transmission as measured in vitro unaffected (Griffiths et al., 2008). Thus it is only the learning-related, long-term changes in transmission that are prevented. The important implication of this selective effect is that the responses of perirhinal neurones to stimuli should continue to be transmitted to other brain regions as normal (with the exception of any change that would otherwise have been produced by learning-related synaptic plasticity). Hence perirhinal non-mnemonic, perceptual functions should be normal after such selective, ‘plasticity lesions’. In particular, it is to be expected that object-related information would be transmitted to the hippocampus for use in an object-in-place task. Thus one interpretation of the lack of impairment of the object-in-place task by interfering with AP2 mechanisms is that the registration (plasticity and storage mechanisms) of where the particular objects had been seen previously are located in the hippocampus and only perirhinal transmission of object identity is necessary for successful performance of the task. An alternative interpretation for the lack of impairment is that the object-in-place task relies on synaptic strengthening rather than weakening processes in perirhinal cortex. The above findings strongly imply that it is possible to impair perirhinal-based recognition memory in the absence of a major perceptual deficit (object-in-place memory is more perceptually demanding than object recognition memory); however, perceptual performance studies have not been conducted after such interventions and there is a need to investigate effects on object-in-place memory at longer delays.

A recent report has suggested that the recognition memory impairment produced by blocking AMPA receptor removal occurs at retrieval (Cazakoff & Howland, 2011). Infusion into perirhinal cortex of Tat-GluA2(3Y), a compound that prevents AMPA receptor removal, produced impairment if infusion was before retrieval, but not if it was before or immediately after acquisition. Whereas a potential effect upon consolidation has a clear theoretical underpinning, an effect upon retrieval remains to be explained.

Intra-perirhinal infusion of an inhibitor of the activity of protein kinase Mzeta (PKMzeta), whose activity is necessary for the maintenance of synaptic strengthening (Sacktor, 2008) impairs object recognition memory when infused after acquisition (Outram, Tinsley, Henley Warburton, & Brown, 2010). In contrast, interfering with PKMzeta in the hippocampus is without effect on single object recognition memory—though it does impair object location memory (Hardt et al., 2010).

### 4.2 Hippocampus

Object recognition memory impairment is produced by blocking nitric oxide synthesis by an infusion into the hippocampus after acquisition, so as to be active during consolidation (Blokland et al., 1998; Furini et al., 2010). Increasing cyclic guanosine monophosphate (cGMP) -related activity by infusion during early consolidation improved rats’ object recognition memory performance (Prickaerts, de Vente, Honig, Steinbusch, & Blokland, 2002). Beta noradrenergic antagonism and inhibition of BDNF function in the CA1 region of the hippocampus after acquisition also impaired memory in the hippocampally-dependent (explore A+B, test A versus C) version of the object recognition task (Furini et al., 2010). The involvement of hippocampal (subfield CA1) cannabinoid receptors in recognition memory consolidation was established using the same task: infusions of the non-selective cannabinoid receptor agonist WIN-55,212-2 or the endocannabinoid membrane transporter inhibitor VDM-11 immediately after acquisition impaired performance of the task after long but not short delays (Clarke et al., 2008).

Using the same hippocampally-dependent version of the object recognition task, impairments in long-term but not short-term memory are produced by hippocampal (CA1) interventions that block protein synthesis in the first few hours following acquisition (Lima et al., 2009), or inhibit the activity of mTOR, a protein kinase involved in the initiation of mRNA translation (Myskiw et al., 2008), or prevent functioning of phosphorylated CREB (Pittenger et al., 2002). A similar impairment was found when protein synthesis was blocked in entorhinal cortex (Rossato et al., 2007). Three of these studies (Lima et al., 2009; Myskiw et al., 2008; Rossato et al., 2007) also found effects upon potential reconsolidation mechanisms when infusions were made immediately after viewing a novel with a familiar object (there was subsequent amnesia relating to both objects). Evidence for involvement of regions of the mouse brain in reconsolidation as well as consolidation mechanisms has been provided by changes in BDNF and egr-1 gene expression in several areas (including perirhinal, entorhinal and prefrontal cortices and hippocampus) after training or reactivation sessions at various time delays following initial acquisition (Romero-Granados et al., 2010). Again, the hippocampally-dependent object recognition memory task was used, together with retraining sessions at long delays.

There are a number of reports of modifications of hippocampal synaptic plastic processes in genetically modified mice that show object recognition memory deficits; however, the genetic modifications are not restricted to the hippocampus and modifications of perirhinal function were not sought (e.g., Jones et al., 2001; Malleret et al., 2001; Mansuy et al., 1998). The studies establish a role for the phosphatase calcineurin (Malleret et al., 2001; Mansuy et al., 1998) and immediate early gene Zif268 (Jones et al., 2001) in object recognition memory, but not which region or which memory phase might be affected. Again, these studies used the hippocampally-dependent version of the object recognition task.

In summary, the large majority of studies that have found an involvement of the hippocampus in the consolidation of recognition memory have used the variant of the object recognition memory task that lesion studies have shown to be dependent on the hippocampus as well as perirhinal cortex (e.g., Clarke et al., 2010; Mansuy et al., 1998; Oh et al., 2010; Pittenger et al., 2002; Barker, *unpublished observations*).

## 5 Recognition memory involving place and time

There have been fewer pharmacological studies of recognition memory for location, temporal order or object-place associations than for object recognition (see Fig. 1B–D for tasks). These studies have investigated the involvement of glutamatergic and cholinergic receptors.

### 5.1 Object location

When a recognition memory judgement depends on spatial memory the hippocampus is critically involved. Thus, lesions in the hippocampus or transection of the fornix produce significant impairments in the object location task as described above (Barker & Warburton, 2011b; Bussey et al., 2000; Ennaceur, Neave, & Aggleton, 1997, though see also Langston & Wood, 2010), yet have no effect on novel object recognition (Barker & Warburton, 2011b; Langston & Wood, 2010). Consistent with this dissociation in the effects of hippocampal lesions, intra-hippocampal infusions of the AMPA receptor antagonist NBQX before acquisition impair object location memory tested following either a 5 min or a 1 h delay, yet have no effect on object recognition memory in rats (Barker & Warburton, 2009). Intra-hippocampal infusions of the NMDA receptor antagonist AP5 before acquisition impair object location memory tested following a 1 h delay. Thus both AMPA and NMDA receptor neurotransmission in the hippocampus are critical for recognition memory for familiar objects presented in a novel location. Selective effects upon acquisition and retrieval have not been investigated. However, temporary inactivation of the hippocampus (using the GABA receptor agonist muscimol) immediately after acquisition produced impairment in mice tested after a 24 h delay, indicating an involvement of the hippocampus in consolidation of object location recognition memory (Oliveira, Hawk, Abel, & Havekes, 2010)—interestingly, this intervention produced an enhancement of object recognition memory.

### 5.2 Object-in-place

**Fig. 5:**
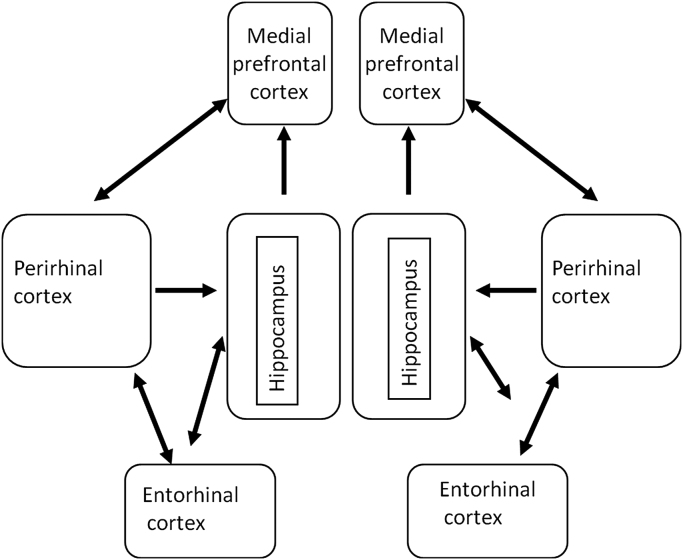
Schematic representation of connections within the perirhinal–hippocampal–medial prefrontal circuit involved in object-in-place and temporal order memory.

Studies employing selective lesions have shown that object-in-place memory depends on multiple brain regions including the perirhinal cortex, medial prefrontal cortex and hippocampus (Barker & Warburton, 2011b; Barker et al., 2007); see Fig. 5. Importantly it has also been established that each of these regions needs to interact with the other two within the neural network for successful task performance. Moreover, insufficient information crosses over between the hemispheres to sustain performance if any two of the structures are removed unilaterally but in different hemispheres. The evidence reviewed above suggests that the contribution of the perirhinal cortex is in the processing and storage of object information, while that of the hippocampus is in spatial processing. As damage to the medial prefrontal cortex impairs object-in-place memory but neither object nor object location recognition memory, the role of medial prefrontal cortex may be in the integration of object and place information necessary to make such recognition memory judgements (Barker et al., 2007). However, it is not necessary to conclude that all the object information and all the spatial information necessary to solve the associative task is held in medial prefrontal cortex (nor as multiple copies in each of the component structures): medial prefrontal cortex may rather hold the associative links (including co-occurrence) between what is stored in the hippocampus and what is held in perirhinal cortex. In such a case, the memory would be held as a distributed engram across the network rather than as, say, an engram confined to medial prefrontal cortex that merely has to be accessed from the other two structures. This issue will only be resolved by eventually determining what type of information necessary to task performance is stored in each of the regions.

#### 5.2.1 Glutamate receptors

Infusion of AMPA receptor antagonists (CNQX or NBQX) bilaterally into either the perirhinal cortex, medial prefrontal cortex or hippocampus impairs object-in-place recognition memory following a 5 min or 1 h delay (Barker & Warburton, 2008a, 2009, 2010). This finding confirms the necessity for excitatory neurotransmission within each of these brain regions for this recognition memory process, and is consistent with the results obtained from lesion studies.

To investigate the necessity for *concurrent* glutamatergic receptor neurotransmission within the perirhinal cortex, medial prefrontal cortex and hippocampus, infusions have been made simultaneously into pairs of different brain regions either unilaterally in the same hemisphere or unilaterally in opposite hemispheres. As with lesions, the expectation is that infusions on the same side will leave performance unimpaired as intervention is restricted to only one hemisphere. Indeed, no impairments have been reported for such single hemisphere infusions, which therefore provide an important control for the effects seen with crossed unilateral infusions that affect both hemispheres. Contrastingly, if two regions need to interact, the crossed infusions will prevent such interaction in both hemispheres and there will be potential behavioural impairment. Indeed, unilateral CNQX infusions into any pair of structures in opposite hemispheres in the hippocampal, perirhinal and medial prefrontal system significantly impair the acquisition and retrieval of object-in-place recognition memory (Barker & Warburton, 2008a, 2009, 2010). Thus concomitant glutamatergic neurotransmission via AMPA/kainate receptors in all of the regions within the circuit is necessary for the performance of an object-in-place task. Moreover, these findings confirm those of lesion studies that possible interhemispheric connections do not rescue performance when there are such crossed interventions.

Infusion of the NMDA receptor antagonist AP5 bilaterally into perirhinal cortex immediately prior to acquisition blocks long-term object-in-place memory tested following a 1 h delay. However, consistent with the findings for non-associative object recognition memory, AP5 infused bilaterally into perirhinal cortex has no effect on shorter-term (5 min delay) object-in-place memory. Additionally, AP5 has no effect on performance when administered before the test phase; thus the impairments are on acquisition rather than retrieval (Barker & Warburton, 2008a). Hence bilateral intra-perirhinal antagonism of NMDA receptors produces the same pattern of delay-dependent acquisition impairments in the object-in-place task as in the object recognition memory task (Barker et al., 2006a).

In contrast to the effects following intra-perirhinal infusion, bilateral infusion of AP5 into the medial prefrontal cortex impairs object-in-place memory following either a short or a longer retention delay. Again, the deficits are only observed when infusions are prior to acquisition and not prior to the test phase. Thus within medial prefrontal cortex, the integration or association of object and place information over a short or long delay relies upon NMDA receptors during acquisition but not retrieval.

The necessity for concurrent NMDA receptor activation within the circuit has been explored in the same way as for AMPA receptors (Barker & Warbuton, 2008a, 2009, 2010). Crossed simultaneous unilateral infusions of AP5 into the hippocampus and medial prefrontal cortex impair both shorter-term and long-term object-in-place recognition memory. In contrast, co-administration of AP5 unilaterally into the perirhinal cortex and medial prefrontal cortex or the perirhinal cortex and hippocampus in opposite hemispheres produces a significant impairment in long-term object-in-place memory, but short-term memory is unaffected. However, infusion of a kainate receptor antagonist unilaterally into the perirhinal cortex combined with a unilateral infusion of AP5 into the contralateral medial prefrontal cortex significantly impairs shorter-term object-in-place memory. Thus shorter-term object-in-place recognition memory is impaired by kainate receptor antagonism just as kainate receptor antagonism in the perirhinal cortex impairs shorter-term object recognition memory. Similarly, broad spectrum antagonism of metabotropic glutamate receptors by infusions before acquisition unilaterally in both perirhinal cortex and medial prefrontal cortex in opposite hemispheres impairs object-in-place recognition memory after a 1 h but not a 5 min delay (Barker & Warburton *unpublished*). Again, this finding is consistent with the effects upon object recognition memory of metabotropic glutamate receptor antagonism within perirhinal cortex (Barker et al., 2006b).

These similar susceptibilities to selective glutamate receptor antagonism in perirhinal cortex at different delays in the two tasks (object and object-in-place recognition memory) suggest that the same perirhinal mechanism is being targeted. In turn, this suggests that perirhinal cortex plays the same information processing role in the two tasks. The most obvious role common to the two tasks is encoding the experiencing of an object. Thus it seems likely that the contribution of the perirhinal cortex to object-in-place recognition memory is to register information about object occurrence. In the hippocampus and medial prefrontal cortex, NMDA antagonism produces impairment in the object-in-place task at both short and long delays, indicating that different underlying mechanisms are involved in these regions. As discussed above, there is much evidence that the hippocampus registers information about spatial locations. The role of the medial prefrontal cortex therefore seems likely to be to associate the hippocampal location with the perirhinal object information.

#### 5.2.2 Cholinergic receptors

A number of studies have explored the effects of cholinergic depletion on variants of object-in-place tasks (Barker & Warburton, 2008b; Easton et al., 2011). Bilateral intracerebral administration of the cholinergic muscarinic receptor antagonist scopolamine into either the perirhinal cortex or medial prefrontal cortex impairs object-in-place memory after either a 5 min or a 1 h delay (Barker & Warburton, 2008b). The impairment is of acquisition and not retrieval. A similar impairment at both delays is produced by unilateral infusions into perirhinal cortex and medial prefrontal cortex in opposite hemispheres, establishing that concurrent muscarinic neurotransmission is required in both regions for performance of the object-in-place task (Barker & Warburton, 2008b). The deficit produced by intra-perirhinal administration of scopolamine is consistent with that for object recognition memory (Tinsley et al., 2011; Warburton et al., 2003); however, the effects of scopolamine on object-in-place task have not been explored at longer delays (>3 h).

### 5.3 Temporal order memory

Fewer studies have explored the pharmacology of temporal order or recency memory, i.e., recognition memory that depends on judging how long ago an item was encountered or the order in which stimuli were presented. Results from studies employing permanent lesions or temporary inactivation through the use of lidocaine have shown that this form of recognition memory is also dependent upon the perirhinal cortex, medial prefrontal cortex and hippocampus (Barker & Warburton, 2011b; Barker et al., 2007; Hannesson et al., 2004a,b; Mitchell & Laiacona, 1998), and that these regions also need to interact (Barker & Warburton, 2011b; Barker et al., 2007; Hannesson et al., 2004b).

#### 5.3.1 Glutamate receptors

Temporal order memory is typically assessed by presenting objects in two sample phases separated by a delay and then, after a further delay, testing the animals ability to differentiate between these two objects i.e., the ‘older’ object that was presented first, and the ‘recent’ object that was presented second. It needs to be noted that there are particular interpretive problems concerning the underlying mechanisms potentially targeted by pharmacological interventions in temporal order tasks. Interventions may target trace strength and/or specific temporal associative mechanisms. Moreover, they may target reconsolidation mechanisms as well as consolidation mechanisms (for the stimuli shown in both sample phase one and two) when interventions are associated with the second sample phase.

The importance of neurotransmission on encoding has been assessed by antagonist administration prior to the presentation of the recent object. If the treatment disrupts memory for the exploration of the recent object, the animals should spend more time exploring the recent than the old object in the test phase (while controls do the reverse because the recently explored object is more familiar). Infusion of CNQX bilaterally into perirhinal cortex prior to the second sample phase results in more exploration of the recent than the old object at test (Barker & Warburton, 2011a). In contrast, infusion of CNQX into medial prefrontal cortex results in equal exploration of the old and recent objects (Barker & Warburton, 2011a). A similar pattern of impairment is produced by infusing AP5 bilaterally into either perirhinal or medial prefrontal cortex; the delay between the second sample phase and the test phase being 3 h (Barker & Warburton, 2011a). Moreover, when CNQX or AP5 are infused unilaterally into perirhinal cortex and medial prefrontal cortex in opposite hemispheres, the impairment is similar to that produced by bilateral medial prefrontal infusions (Barker & Warburton, 2011a). Thus both AMPA/kainate and NMDA receptors are necessary for acquisition of temporal order memory tested after a 3 h delay. Administration of CNQX but not AP5 prior to the test phase impairs retrieval (Barker & Warburton, 2011a). Thus for temporal order memory, as for the object and object-in-place recognition memory, perirhinal NMDA receptors are critical for acquisition but not retrieval when tested after a long delay, while perirhinal AMPA receptor transmission is essential for both acquisition and retrieval at short or long delays.

#### 5.3.2 Cholinergic receptors

Muscarinic receptor antagonism with bilateral infusions of scopolamine into the perirhinal or medial prefrontal cortex produces similar deficits in the acquisition of temporal order memory as does AP5 or CNQX administration (Barker & Warburton, 2011a). Infusion into the perirhinal cortex prior to the second sample phase increases exploration of the recent object compared to the old object during the test phase, whereas infusion into the medial prefrontal cortex results in equal exploration of the old and recent objects. When scopolamine is infused unilaterally into perirhinal cortex and medial prefrontal cortex in opposite hemispheres, the impairment is similar to that produced by bilateral medial prefrontal infusions (Barker & Warburton, 2011a).

#### 5.3.3 Overview for temporal order memory

NMDA glutamatergic or muscarinc cholinergic receptor antagonism within the perirhinal cortex and medial prefrontal cortex produce region specific deficits in acquisition of temporal order memory. With intra-perirhinal infusions before the recent object is explored, the recent object is treated as if novel, the same impairment as is found for object recognition memory. Thus the most plausible explanation is that the impaired process is indeed the same, i.e., disruption of registration of the experiencing of the object or the recent object (Barker & Warburton, 2011a,b). In contrast, NMDA and muscarinic receptor blockade in the medial prefrontal cortex before the second sample phase results in equal exploration of the recent and the old object, suggesting that both objects are regarded as equally familiar. This suggests that what has been lost is information about the time elapsed since exploration, or the order of object presentation, or memory of the object sequence (Barker & Warburton, 2011a). Thus the impairment appears to be in temporal rather than object information.

The temporal order recognition memory impairments resulting from unilateral pharmacological disruption of the perirhinal cortex and medial prefrontal cortex in opposite hemispheres indicate that these regions function as an integrated neural circuit, in confirmation of the ablation findings (Barker & Warburton, 2011a,b; Hannesson et al., 2004a). Interestingly, the resulting impairment is similar to that seen following infusions into the medial prefrontal cortex, i.e., there is no discrimination between the old and recent objects at test. Accepting the above suggestions as to roles of the regions, in one hemisphere the output of the fully functional perirhinal cortex (both objects familiar) goes to the compromised medial prefrontal cortex (compromised time-signature). In the other hemisphere, the output of the compromised perirhinal cortex (recent is novel), goes to the fully functional medial prefrontal cortex; however, the data indicate that this signal is insufficient to drive greater exploration of the recent than the old object when it is in conflict with the signal from the other hemisphere.

### 5.4 Summary

In summary, pharmacological studies of other types of recognition memory have confirmed the results of lesion studies that establish the importance of regions other than the perirhinal cortex for these other types of recognition memory. Thus object location recognition memory is dependent upon the hippocampus and does not require the involvement of perirhinal cortex as the identity of the moved object is not required for determining that a new location has been occupied. Object-in-place and temporal order recognition memory depend upon a neural circuit involving the perirhinal cortex, hippocampus and medial prefrontal cortex. Each component region of this circuit is essential, as is their interaction. Nevertheless, the indications are that the different components perform different functions. In confirmation of the work on object recognition memory, perirhinal cortex appears to provide information about objects and their familiarity, while the hippocampus provides spatial information and the medial prefrontal cortex is important for forming associational connections and for temporal information. Moreover, the roles of each region have been demonstrated to encompass both acquisition and retrieval, with acquisition being dependent upon AMPA and NMDA glutamatergic and also muscarinic cholinergic neurotransmission.

## 6 Conclusions

The reviewed pharmacological studies provide a large body of evidence supporting the essential role of the perirhinal cortex in rodent object recognition memory. These studies therefore support the consensus view from human work of the central importance of perirhinal cortex to recognition memory (Brown et al., 2010; Eichenbaum et al., 2007; Mayes et al., 2007; Montaldi & Mayes, 2010; Montaldi et al., 2006; Squire et al., 2007). However, the rodent pharmacological studies allow the establishment of an essential role for the perirhinal cortex in the acquisition, the consolidation and the retrieval of such memory. Moreover, the necessity to rat long-term recognition memory of intracellular signalling mechanisms related to synaptic modification within perirhinal cortex establishes a central role for the region in the information storage underlying such memory. Perirhinal cortex is thereby established as a storage site rather than solely a processing station.

Pharmacological studies are providing increasingly detailed evidence concerning both the neurotransmitter systems and the underlying intracellular mechanisms involved in recognition memory processes. In a few cases (for example cholinergic antagonists), rat findings have parallels in human studies though, because administration in the human case is necessarily systemic, the rat findings add important data concerning the locus of action of the drugs. The rodent studies have provided evidence in support of synaptic weakening as a major synaptic plastic process within perirhinal cortex underlying object recognition memory and confirmatory evidence that there is more than one synaptic plastic process involved. Challenges have been provided to the view that perirhinal memory impairments are explicable as merely some by-product of its perceptual functions or solely explicable by increased susceptibility to interference: it is also necessary to understand the underlying storage mechanisms.

Rodent pharmacological studies are also providing new evidence concerning the detailed roles of other regions, including the hippocampus and the medial prefrontal cortex in different types of recognition memory tasks that include a spatial or temporal component. In so doing, they also provide information concerning the contribution of perirhinal cortex to such tasks. These studies hence help to inform the debate about whether the role of perirhinal cortex can be separated from that of the hippocampus (Aggleton & Brown, 2006; Eichenbaum et al., 2007; Montaldi & Mayes, 2010; Montaldi et al., 2006; Murray et al., 2007; Norman, 2010; Squire et al., 2007; Squire & Wixted, 2011; Vann et al., 2009; Vann & Albasser, 2011). To date it appears from the rat findings that the contribution of perirhinal cortex to associative and temporal order memory reflects that in simple object recognition memory, namely that perirhinal cortex provides information concerning objects and their prior occurrence (novelty/familiarity).
